# *Gordonia* species as a rare pathogen isolated from milk of dairy cows with mastitis

**DOI:** 10.1038/s41598-022-09340-4

**Published:** 2022-04-11

**Authors:** Jaroslav Bzdil, Sona Slosarkova, Petr Fleischer, Jan Matiasovic

**Affiliations:** 1 Department of Special Microbiology, State Veterinary Institute Olomouc, 779 00 Olomouc, Czech Republic; 2 Department of Infectious Diseases and Preventive Medicine, Veterinary Research Institute, 621 00 Brno, Czech Republic

**Keywords:** Applied microbiology, Bacteria, Infectious-disease diagnostics, DNA sequencing, Bacterial genes

## Abstract

While *Gordonia* species have long been known to cause severe inflammation in humans, the pathogenic effects of *Gordonia* species in veterinary medicine have rarely been described. Between 2010 and 2019, we collected microorganisms of the genus *Gordonia* isolated from milk samples from dairy cows with mastitis. We describe the growth properties of these microorganisms and their prevalence, virulence factors and susceptibility to antimicrobial agents. From 31,534 quarter milk samples processed by standard culture methods, 27 isolates of *Gordonia* species (0.086% prevalence) were identified by a molecular phenotyping method. The isolates originated from 17 farms in 12 districts of the Czech Republic. Twenty-one isolates were tested for susceptibility to 7 antimicrobials by the disc diffusion method. Notably, 100% of these isolates were susceptible to streptomycin and neomycin, 85.7% to cefovecin and tetracycline, 76.2% to penicillin G, 47.6% to trimethoprim/sulfamethoxazole and 0% to clindamycin. The species was determined to be *Gordonia paraffinivorans* by whole genome sequencing for 9 isolates (from 8 farms in 7 districts). These isolates showed the highest similarity to two reference strains from the environment. In all these isolates, we identified genes encoding virulence factors that are very similar to genes encoding virulence factors expressed in *Mycobacterium tuberculosis* and *Mycobacterium smegmatis*. However, genome analysis revealed 61 unique genes in all 9 sequenced isolates.

## Introduction

Members of the genus *Gordonia* (order *Actinomycetales*, suborder *Corynebacterineae*) are aerobic, catalase-positive, gram-positive-to-gram-variable, slightly acid-fast, nonmotile, nocardioform actinomycetes. *Gordonia* species do not generate spores^1^. Some of these microorganisms were initially classified as rhodococci, and *Gordonia* species are rarely reported as causative agents of diseases in humans^2,3^. Understanding of their medical significance and pathogenicity among the community of medical and veterinary professionals is still limited. The genus *Gordonia* (*G.*) currently includes 40 bacterial species^4^ that are mainly found in the environment, particularly in the soil, but have also been isolated from animals, for example, from the digestive tract of mammals^2^. Some members of this genus are utilized in the bioremediation or biodegradation of pollutants and in the pharmaceutical industry^5,6^. *Gordonia bronchialis* has been shown to positively modulate immunity in animals such as rats, dogs and mice and in fish and shrimp in aquaculture^7^.

*Gordonia paraffinivorans* has been found in hydrocarbon-contaminated environments such as oil fields^8,9^. It has also been isolated from compost after selective propagation in a medium with hydrocarbons as carbon sources^10^ and together with other *Gordonia* species in biofilms in milking machines^11^. Simultaneously with our study, *G. paraffinivorans* was identified as a mastitis-causing pathogen of dairy cows in several regions in two other Central European countries, Hungary and Germany, in a thesis in Hungarian^12^ and in a German language journal for practising veterinarians^13^, respectively. In both studies, the species was verified via 16S rDNA sequencing. The isolations of pure cultures of this causative agent from 21 quarter milk samples from 14 cows with signs of mastitis (and some even with microscopic evidence of infection with *Gordonia* species) or with high somatic cell count history (reason for culture) on 9 farms out of a total of 708,330 processed milk samples in the period between 2015 and 2019 was described. In some cows, there was repeated isolation from the same quarter after three weeks^13^.

Other species, such as *G. bronchialis* and *Gordonia sputi*, are associated with diseases, particularly in immunocompromised human patients^1,14^. Furthermore, five cases of human septicaemia caused by *Gordonia* sp. have been described^3^. Only two cases of human granulomatous mastitis in immunocompetent patients (induced by *Gordonia terrae* and *G. bronchialis*) have been described to date^15,16^.

Other authors have reported that *Gordonia aichiensis, Gordonia amicalis, Gordonia arai, G. bronchialis, Gordonia effusa, Gordonia otitidis, Gordonia polyisoprenivorans, G. sputi* and *G. terrae* cause not only skin and soft tissue infections but also diseases of the bones and joints^2,17,18^. In animals, only solitary cases, e.g., lymphadenitis in pigs with isolation of *G. sputi* ^19^ and in zebu with isolation of *Gordonia sinesedis*^20^, have been reported. Good susceptibility (low minimum inhibitory concentrations (MICs) for penicillin, clindamycin, tetracycline, streptomycin, trimethoprim/sulfamethoxazole, etc.) were noted in a few isolates of *G. terrae* and *G. sputi* during antimicrobial susceptibility testing for 20 antimicrobial agents, whereas high MICs were observed for nitrofurantoin and quinupristin-dalfopristin^21^.

The aim of this study is to report on our findings regarding *Gordonia* sp. in the milk of dairy cows with signs of mastitis to briefly describe their identification, growth characteristics, prevalence, genes encoding possible virulence factors and susceptibility to antimicrobial agents and to compare our results with the data in the literature on *Gordonia* spp. isolated from clinical material.

## Results

### Sample cultivation, confirmation and characterization of isolates

**Figure 1:**
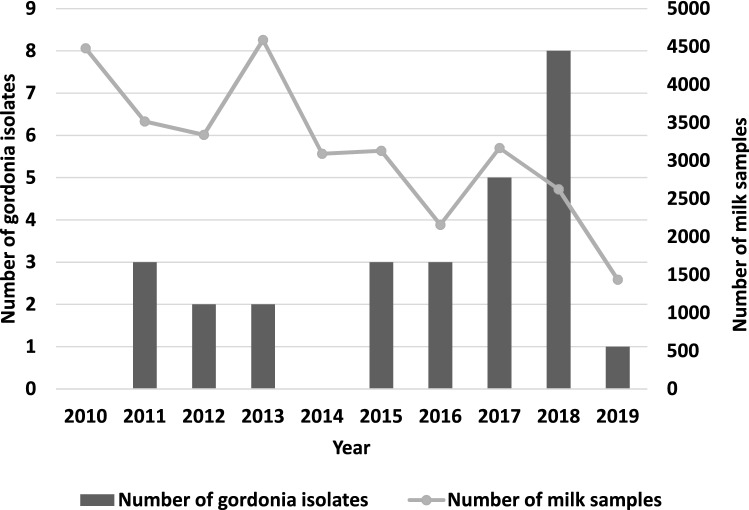
Number of bacteriologically examined bovine mastitis milk samples (n = 31,534) and *Gordonia* sp. isolates (n = 27) per year.

Out of 31,534 quarter mastitis milk samples, a total of 27 pure cultures of *Gordonia* sp. were isolated from 27 dairy cows over a period of ten years. These 27 samples constituted 0.086% of the samples, with detection of *Gordonia* sp. as the dominant pathogen. Figure 1 shows the numbers of annually examined milk samples and *Gordonia* sp. isolates. The annual prevalence was 0–0.3%. Table 1 provides anamnestic and geographic data for individual isolates. The isolates originated from 17 (9.7%) farms in 12 (20.3%) districts that provided milk samples, across five regions (with a total of 26 districts) of the Czech Republic. *Gordonia* sp. was isolated across all seasons.

**Table 1:** Anamnestic data for *Gordonia* sp. isolates (n = 27) from mastitis milk of dairy cows.

Isolate	Date of sample acceptance	Farm	Number of samples examined (portion with *Gordonia* sp.; %)	District^a^ of CZ^b^	Region of CZ	Genotyping code
1; 2	2011-11-29	1	39 (5.1)	FM	Moravian-Silesian	F10; –
3	2011-12-06	2	41 (2.4)	OL	Olomouc	F9
4	2012-07-31	3	35 (2.9)	SY	Pardubice	G2
5; 6	2013-03-28	3	13 (15.4)	SY	Pardubice	–; G3
7	2013-07-23	4	10 (10.0)	OL	Olomouc	F12
8	2015-09-21	5	6 (16.7)	PR	Olomouc	G4
9	2015-10-16	6	35 (2.9)	SY	Pardubice	–
10	2015-10-23	7	10 (10.0)	NJ	Moravian-Silesian	G5
11	2016-02-13	8	3 (33.3)	PV	Olomouc	G1
12	2016-10-10	9	8 (12.5)	UH	Zlín	F11
13	2016-10-25	4	10 (10.0)	OL	Olomouc	–
14	2017-04-03	10	5 (20.0)	BO	South-Moravian	–
15	2017-07-03	8	13 (15.4)	PV	Olomouc	–
16	2017-07-31	9	26 (3.8)	UH	Zlín	–
17; 18	2017-10-09	11	16 (12.5)	PR	Olomouc	–; –
19	2018-02-08	9	9 (11.1)	UH	Zlín	–
20	2018-03-06	12	4 (25.0)	HO	South-Moravian	–
21; 22; 23	2018-06-05	13	10 (30.0)	UH	Zlín	–; –; –
24	2018-09-04	14	1 (100.0)	SY	Pardubice	–
25	2018-10-17	15	1 (100.0)	SU	Olomouc	–
26	2018-11-14	16	10 (10.0)	KM	Zlín	–
27	2019-10-23	17	1 (100.0)	BV	South-Moravian	–

^a^Official abbreviation of the district name; ^b^CZ = Czech Republic.

Due to the primarily microbiological focus of the work, a detailed clinical history was retrospectively obtained for only 12 isolates, i.e., for 12 cows, 10 were diagnosed with parenchymatous mastitis, 8 of them with acute parenchymatous mastitis, i.e., a severe form of clinical mastitis (severity score: 3), and two with recurrent chronic mastitis.

**Figure 2:**
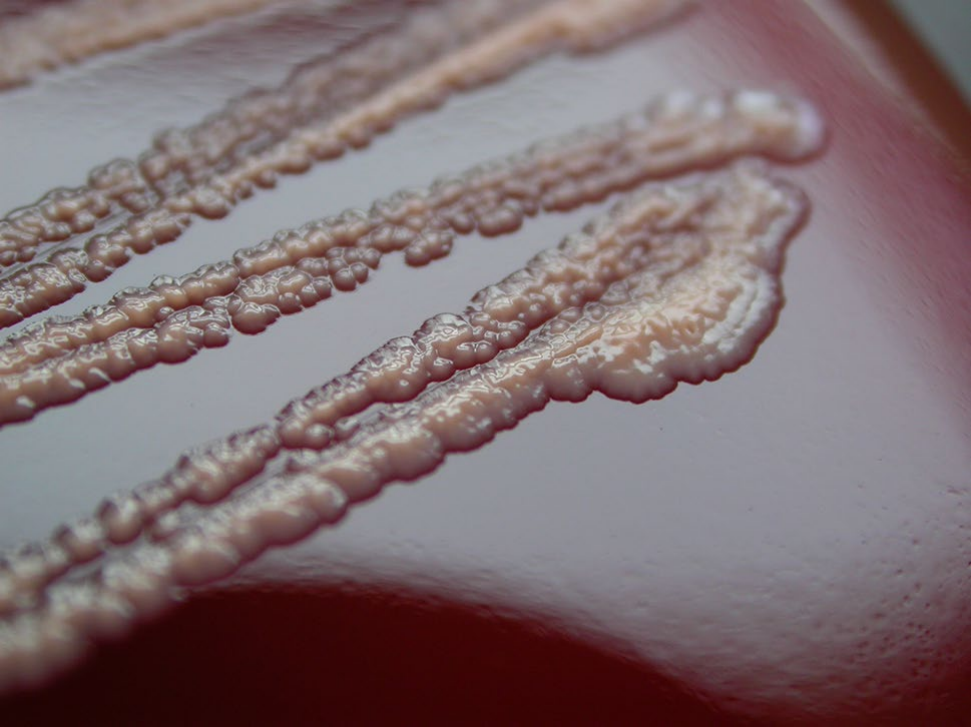
*Gordonia paraffinivorans* growth on a Meat Peptone Blood Agar plate after 72 h of incubation of pure culture isolated from primary culture of a milk sample.

**Figure 3:**
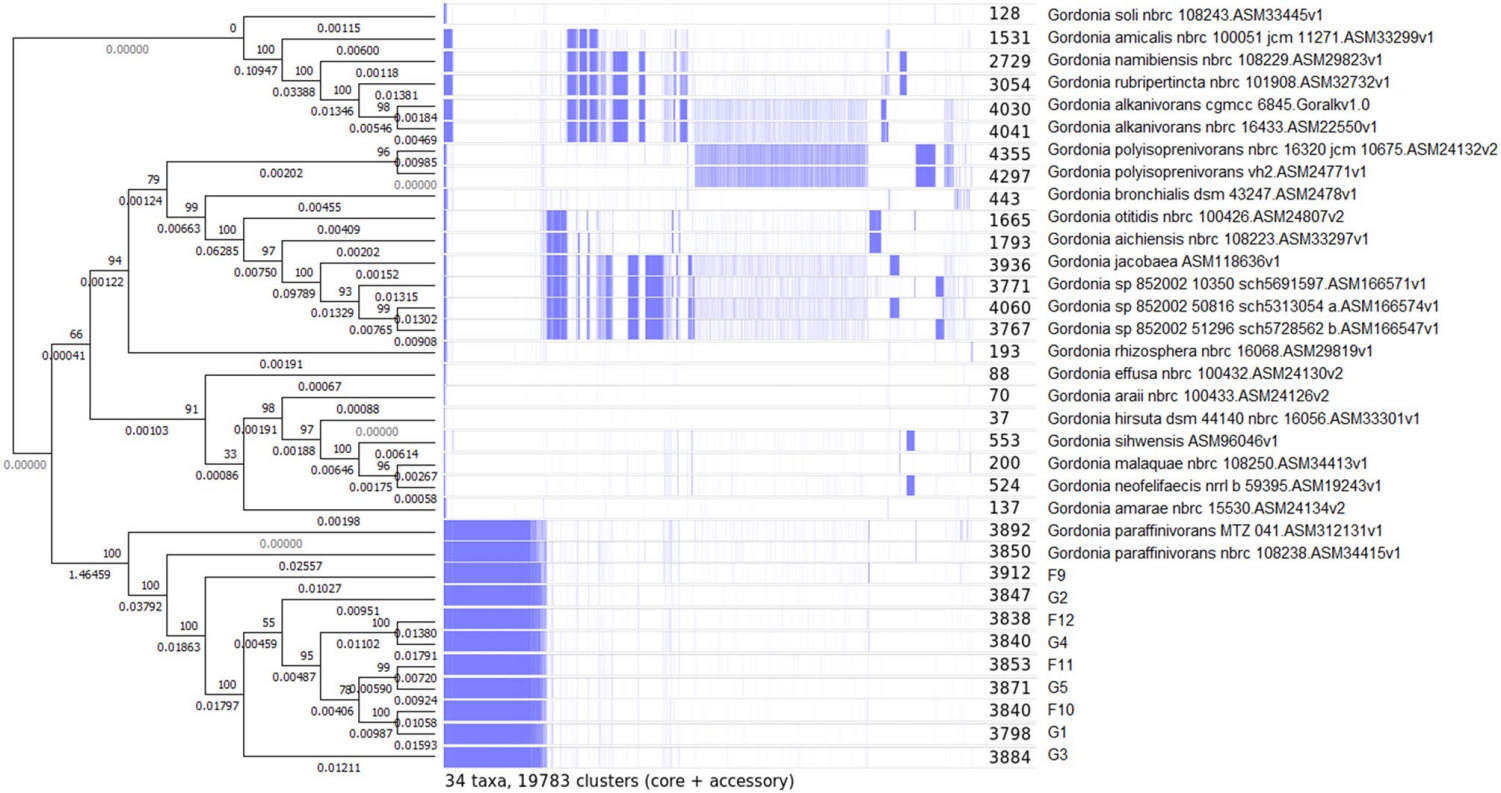
Genetic relationship of *Gordonia paraffinivorans* mastitis isolates (Field *Gordonia paraffinivorans* mastitis isolates (n = 9) are marked as F9–F12 and G1–G5. Neighbor-joining tree was constructed on the matrix of presence—absence of genes. Numbers in nodes represents bootstrap values, branch length represent relative genetic distance. In the Roary matrix (middle section), the presence of gene common to at least two genomes is depicted by blue stripe. Number of common genes in genome is shown next to genome identification.) and *Gordonia sp*. reference genomes.

After 24 h of incubation, the colonies of *Gordonia* sp. on Meat Peptone Blood Agar (MPBA) were 0.5–3.0 mm in diameter, they were non-haemolytic, catalase positive, oxidase negative, small, slightly convex or flat, rough and dull with uneven margins; they looked pale pink, orange and, later, reddish coloured (Fig. 2) and emitted various rotting odours (stronger after prolonged incubation). Semiquantitative assessments showed moderate (+++) or heavy (++++) growth. Using MALDI-TOF MS, all isolates were identified as *Gordonia rubripertincta*, with an identification score of 1.723–2.319 (probable genus identification, probable species identification; Supplementary file 1). For 9 selected isolates (from 8 farms in 7 districts; with an identification score of 1.781–2.162), species identification was performed by whole-genome sequencing using Illumina technology. These 9 isolates showed the closest relatedness to *G. paraffinivorans* NBRC 108238 (Fig. 3). Five genes encoding virulence factors similar to the virulence genes of *Mycobacterium tuberculosis* were found in the genome of all isolates sequenced in the present study, with the exception of 1 gene in 2 isolates in all cases*.* These virulence factor genes were also present in both reference strains of *G. paraffinivorans* isolated from the environment (Table 2).

**Table 2:** Virulence factor genes present in *Gordonia paraffinivorans*^a^ according to virulence factor database.

Virulence factor gene^†^/Isolate^‡^	F9	F10	F11	F12	G1	G2	G3	G4	G5	NBRC 108238	MTZ 041
icl, Isocitrate lyase [YP_177728, *Mycobacterium tuberculosis* H37Rv]	+	+	+	+	+	+	+	+	+	+	+
relA, Probable GTP pyrophosphokinase [NP_217099, *Mycobacterium tuberculosis* H37Rv]	+	+	+	+	+	+	+	+	+	+	+
ideR, Iron-dependent repressor and activator [NP_217227, *Mycobacterium tuberculosis* H37Rv]	+	+	+	nd	+	+	+	+	+	+	+
mbtH, putative protein [NP_216893, *Mycobacterium tuberculosis* H37Rv]	+	+	+	+	+	+	+	nd	+	+	+
mbtL, Acyl carrier protein [NP_215860, *Mycobacterium tuberculosis* H37Rv]	+	+	+	+	+	+	+	+	+	+	+

^a^Mastitis isolates (n = 9) and NBRC 108238 and MTZ 041 strains; ^**†**^Only virulence genes with percentage of coverage greater than 40% and percentage of identity greater than 75% to virulence factor database genes are present; *+* gene present; *nd* not detected; ^**‡**^Field *G. paraffinivorans* mastitis isolates are marked as F9–F12 and G1–G5.

### Genome fragments unique to *Gordonia paraffinivorans* isolates from cows with mastitis

Detailed comparison of our nine *G. paraffinivorans* isolate sequences to *G. paraffinivorans* reference NBRC 108238 and MTZ 041 genomes revealed a couple genome fragments common to all nine selected mastitis isolates but not present in the environmental strains. Fourteen of them were larger than 1000 bp (Supplementary file 2). The largest unique fragment consisted of 40,400 bp with 37 annotated coding sequences. In the genome fragment contig00009.v75000-79000, two copies of a gene encoding mammalian cell entry protein (locus_tag = "c00009_00002" and locus_tag = "c00009_00003") were found to be similar to a gene encoding mammalian cell entry protein from *Mycobacterium smegmatis* str. MC2 155, but similar genes were also present in the NBRC 108238 and MTZ 041 genomes.

### Genes unique to *Gordonia paraffinivorans* from the environment or mastitis isolates

Comparison of gene annotation of each of our tested isolates revealed the presence of 3,522 genes common to all *G. paraffinivorans* mastitis isolates and to NBRC 108238 and MTZ 041. Another 1,694 “cloud” genes were present in at least one isolate. In NBRC 108238 and MTZ 041, another 70 genes were present (Supplementary file 3), most of which were hypothetical genes with unknown function. Another 61 genes were present in all tested mastitis isolates (Supplementary file 4) but not in NBRC 108238 or MTZ 041. Half of them are located on a unique 40,400 bp genome fragment, and most of the genes present on this fragment are involved in cholesterol degradation. The rest of the genes unique to our *G. paraffinivorans* isolates are mostly hypothetical with unknown function.

### Antimicrobial susceptibility

The results of susceptibility testing of 21 isolates to 7 antimicrobials are shown in Table 3. All tested isolates (100%) were resistant to clindamycin and susceptible to neomycin and streptomycin. There were both susceptible and resistant isolates to each of the other four antimicrobials.

**Table 3:** Susceptibilities of *Gordonia* species mastitis isolates (n = 21) to antimicrobial agents.

Antimicrobials	Disc content (µg)	Susceptible isolates	
		(No)	(%)
Penicillin G	10	16	76.2
Cefovecin	30	18	85.7
Streptomycin	10	21	100
Neomycin	30	21	100
Clindamycin	2	0	0
Tetracycline	30	18	85.7
Cotrimoxazole	25	10	47.6

Resistance profiles for *Gordonia* species mastitis isolates are shown in Table 4. Our frequent patterns of antimicrobial susceptibility were resistance only to clindamycin (n = 7) followed by resistance to clindamycin and trimethoprim/sulfamethoxazole (n = 6). Five isolates were considered multidrug resistant (resistance to ≥ 3 groups of antimicrobials).

**Table 4:** Resistance profiles for *Gordonia* species mastitis isolates (n = 21).

Frequency of resistance by		Phenotype of resistance	Number of isolates	Sum of multidrug-resistant isolates
Active substance	Antimicrobial group			
1	1	CLI	7	
2	2	CLI, COT	6	
2	2	CLI, TET	2	
3	2	PNC, CFV, CLI	1	0
3	3	PNC, CLI, COT	2*	
3	3	CLI, TET, COT	1*	
4	3	PNC, CFV, CLI, COT	2*	5

*CLI* clindamycin, *COT* sulfamethoxazole/trimethoprim, *TET* tetracycline, *PNC* penicillin G, *CFV* cefovecin; *Multidrug resistant isolates, i.e. resistance to ≥ 3 groups of antimicrobials.

A number of multidrug resistance genes with high similarity to genes in a number of other *Gordonia* species (*Stp*: 98–100% coverage, 80–86% identity; *EbrA*: 93–98% coverage, 72–78% identity; *MdtD*: 92–97% coverage, 75–87% identity; *EmrY*: 89% coverage, 73% identity only to *G. rubripertincta* and *Gordonia jinghuaiqii* genes) were found in all nine sequenced isolates, as well as in the NBRC 108238 and MTZ 041 strains. The same result applies for tetracycline resistance protein class C and the daunorubicin/doxorubicin resistance genes *DrrA*, *DrrB* and *DrrC* (51–100% coverage, 71–95% identity to genes in number of other *Gordonia* species). One of the *Stp* gene variants found in all compared *G. paraffinivorans* genomes had 66% nucleotide similarity to the *lmrC* gene associated with lincomycin resistance in *Streptomyces lincolnensis*, a lincomycin producer.

## Discussion

Hungarian and German studies^12,13^ and our results show that clinical lesions attributable to the pathogenic effects of *Gordonia* sp. can also be encountered in numerous animal cases, contrary to earlier reports with only one case per study^19,20^. Out of the 40 known species of the *Gordonia* genus^4^, only one species, *Gordonia paraffinivorans,* was detected in mastitis cow milk in 27 samples in the Czech Republic between 2010 and 2019 and, in parallel, in 22 samples in Hungary between 2010 and 2013^12^ and in 21 samples in Germany between 2015 and 2019^13^. The difference in prevalence in these Czech and Germany sets (0.086% versus 0.003%) of milk samples is relatively large, i.e., the value is almost thirty times lower in the whole German set (n = 708,330), which probably reflects a different sampling strategy consisting of predominantly preventive examinations/cultures. Taking into account only German samples from cows with udder health disorders (n = 223,008; *G. paraffinivorans* was isolated from these samples)^13^, the prevalence (0.0094%) of *G. paraffinivorans* infection was nine times lower. Our annual prevalence (0–0.3%) and portions of samples with *Gordonia* sp. delivered on a given day from a farm (2.4–100%; Table 1) were probably influenced by a broader change during this decade that many farms in the Czech Republic have introduced rapid on farm culture testing to diagnose mastitis pathogens and only sent atypical cases to the laboratory.

The detections of *Gordonia* spp. in human patients have been substantially more diverse. Nine *Gordonia* spp. have been isolated from pathological processes following cardiothoracic surgery as well as from lesions of the skin, soft tissues, bones and joints^2,17,21^, and these microorganisms have also been isolated from biological materials collected from septic patients^3^. Two *Gordonia* spp. have also been identified as causative agents of two cases of human mastitis^15,16^.

It follows that the diversity of pathogenic *Gordonia* spp. and their sources are much more varied in human medicine than in the veterinary sphere. This can be partly explained by the fact that modern diagnostic methods (MALDI-TOF MS, PCR) have arrived into wide or routine use in veterinary diagnostic laboratories later than in human medicine. Furthermore, the differences between human and animal immunity and their changes may also play a role. In a number of reported *Gordonia* infections^5,22,23^, immunocompromised status was the major risk factor, but infections were also reported in 15 immunocompetent patients who were mostly elderly persons with major primary comorbidities or who underwent exacting sternotomy-type surgery^18^. In contrast, farm animals in particular are not allowed to die of old age (especially if seriously ill), and they do not commonly undergo extensive surgery. In dairy cows, a significant risk of mammary gland infection is generally a consequence of high milk yields^24^ and intensive dairy farming (carried out particularly in Europe and North America). Mastitis is the most economically important disease in dairy cows worldwide.

Because *Gordonia* sp. isolated in our study was detected on 17 farms (also simultaneously in several cows and repeatedly on the same farms) in almost half of the districts of the large main catchment area in the eastern part of the Czech Republic, a wide area of occurrence of this pathogen can be assumed. The German study isolated *G. paraffinivorans* on nine dairy farms in the region of Saxony, i.e., the federal state next to the northwest border of the Czech Republic. The Hungarian study isolated *G. paraffinivorans* on five dairy farms in four regions of northwestern Hungary. Therefore, it is probably a pathogen associated with dairy cow mastitis not only in the Czech Republic but also in a substantial part of Central Europe, albeit in sporadic cases.

From a genetic point of view, the nine *Gordonia* sp. isolates sequenced in our study were most closely related to *Gordonia paraffinivorans* NBRC 108238 (Fig. 3), which was isolated from oil-contaminated water, and MTZ 041 (isolated from compost). However, five genes encoding virulence factors similar to virulence genes of *Mycobacterium tuberculosis* were found in the genome of the isolates from mastitis cases and both environmental reference strains^25^. This coincidence suggests that *G. paraffinivorans* has the potential to cause disease as an opportunistic pathogen. Furthermore, in contrast to the two copies of the mammalian cell entry protein gene in the reference genome of *G. paraffinivorans* NBRC 108238, mastitis isolates tested in our laboratory contained a unique genome fragment with another two mammalian cell entry protein-like genes. All isolates tested in the present study contained 61 unique genes that were not present in NBRC 108238 or MTZ 041. Almost half of these genes are hypothetical, and their function is unknown, but approximately one-third of them are involved in the cholesterol degradation pathway. This suggests a nonaccidental occurrence of these isolates in mastitis samples and a probable adaptation of these strains to the internal environment of the mammalian body as a minor environmental mastitis pathogen, which is in accordance with the other two studies^12,13^.

A potential explanation for the discrepancy in species identification between MALDI-TOF MS and whole genome sequence analysis is the incompleteness of the MALDI-TOF MS database, which currently contains a spectrum of 6 *Gordonia* spp. only (*G. aichiensis*, *G. alkanivorans*, *G. bronchialis*, *G. rubripertincta*, *G. sputi* and *G. terrae*), but *G. paraffinivorans* spectra are still missing there.

Differences in antimicrobial susceptibility were observed in veterinary isolates tested in our study as well as in human clinical isolates of *Gordonia* spp. Because of the various susceptibility testing methods used in different studies, the examination of different *Gordonia* spp. and the limited number of cases tested in the studies, it is difficult to adopt a clear position on this issue. Nevertheless, in accordance with the detected susceptibility to streptomycin and neomycin in all isolates tested in our study, human clinical isolates of *Gordonia* were also susceptible to aminoglycosides, whereas there was a discrepancy in the results obtained by susceptibility testing results for penicillin and trimethoprim/sulfamethoxazole reported in different papers^18,26,27^. Regarding clindamycin, there is a study in which *Gordonia* spp. (specifically *G. terrae* and *G. sputi*) were susceptible, i.e., had low minimum inhibitory concentrations^27^, and a study in which a clindamycin-resistant isolate of *G. bronchialis* was found^14^. The genomes of all *G. paraffinivorans* isolates from mastitis cases and the strains NBRC 108238 and MTZ 041 from the environment contain a variant of the multidrug resistance *Stp* gene similar to the lincomycin resistance gene of *Streptomyces lincolnensis*, a lincosamide antibiotic producer^28^. The resistance of all 21 isolates tested for clindamycin susceptibility in our study can also be associated with the fact that clindamycin is a lincosamide antibiotic, whose other representatives, especially lincomycin, were previously used in the Czech Republic mainly to treat severe mastitis, whether directly infused into the udder or given as an injection, or was used as a first-option antibiotic. In our intensive recent study of antimicrobial resistance of mastitis-causing gram-positive bacteria from the same intake area/catchment area, clindamycin was the antimicrobial with the third highest rate of resistant isolates of *Streptococcus* sp.^29^. A certain parallel can also be seen with trimethoprim/sulfamethoxazole, which are representatives of the group of potentiated sulfonamides previously used (even in the intramammary form) for the treatment of mastitis and many other primary and secondary bacterial infections of dairy cows in the Czech Republic. Multidrug resistance was detected in 23.8% of our isolates of *Gordonia* sp., which is a lower frequent rate than that observed in the most prevalent gram-positive bacteria causing mastitis in the Czech Republic (*S. uberis*), with 38.7% of its isolates^29^.

This is one of first studies reporting isolation of *Gordonia* species from numerous animal cases. Expected reports of *Gordonia* sp. from other veterinary laboratories may primarily reflect improved diagnostics. At the same time, mastitis caused by *Gordonia* sp. can probably be considered a small part of the global shift in the spectrum of mastitis agents in intensively reared dairy cows, from the original predominance of contagious agents to environmental agents^12^.

## Conclusion

In the present study, we identified bacteria of the genus *Gordonia* as mastitis pathogens in dairy cattle in a large area of the Czech Republic over a decade, albeit in sporadic cases. Whole genome sequencing showed a very close relationship of the isolates tested in our study with reference strains of *Gordonia paraffinivorans.* However, a unique cluster of genes was found in all mastitis isolates. Antimicrobial susceptibility rates of the twenty-one tested isolates revealed high resistance to trimethoprim/sulfamethoxazole and low resistance to some beta-lactam antibiotics and tetracycline. All tested isolates were resistant to clindamycin and susceptible to neomycin and streptomycin. Five isolates were considered multidrug resistant.

## Materials and methods

### Milk samples

A total of 31,534 quarter milk samples were collected from dairy cows with mastitis. In a total of 59.3% samples (n = 18,705) were specified that they are from dairy cows with clinical mastitis and in a total of 22.7% of samples (n = 7153) were specified that they are from dairy cows with subclinical mastitis. In a total of 18% of samples, the type of mastitis was not indicated (n = 5676; 18.0%). The samples were delivered on a voluntary basis by local veterinarians from 175 farms in 59 districts of the Czech Republic (CZ) between January 2010 and December 2019 to the pick-up points of the State Veterinary Institute in Olomouc. The samples were transported in cool boxes at 4 °C. The numbers of examined samples in individual years of the monitoring period are shown in Fig. 1.

### Isolation and identification of bacteria

The samples were subjected to conventional bacteriology (cultivation, isolation and identification of the agents). All milk samples were inoculated onto Meat Peptone Blood Agar (MPBA) (TRIOS, spol. s r.o., Prague, CZ) and incubated aerobically at 37 ± 1 °C for 42–48 h. The growth of individual cultures was evaluated semiquantitatively, and the results were reported as sporadic (+) to heavy (++++) growth. On plates with mixed bacterial cultures, i.e., containing 2 or maximally 3 different bacterial species, the most frequent colony-forming agent was regarded as the dominant species, i.e., as the dominant pathogen^29^. Plates containing more than three different bacterial species were classified as contaminated without further identification of bacterial species.

The isolation of suspected colonies of *Gordonia* sp. from monocultures or dominant cultures was continuously performed on MPBA according to standard operating procedures. Pure cultures of *Gordonia* sp. were identified using phenotypic molecular mass spectrometry, i.e., matrix-assisted laser desorption/ionization coupled to time-of-flight mass spectrometry (MALDI-TOF MS) using a Microflex LT System spectrometer (Bruker Daltonik GmbH & Co. KG, Bremen, Germany), based on proteomics analyses, and MALDI Biotyper software MBT Compass 4.1 (Bruker Daltonik GmbH & Co. KG, Bremen, Germany)^30^. The isolates were stored in microtubes of the ITEST KRYOBANKA B system (ITEST plus, s.r.o., Hradec Králové, CZ) at − 72 °C.

### Whole genome sequencing

Nine selected isolates of *Gordonia* sp. collected in 2011–2016 (one per farm, with one exception of two samples from one farm but eight months apart) from randomly selected cows were sequenced to obtain their whole genome sequences. The number of selected isolates was limited by the cost per sequencing. Genomic DNA of these *Gordonia* sp. isolates (further referred to as F9–F12 and G1–G5) was isolated from colonies grown on MPBA plates using a Qiagen DNeasy Blood and Tissue Kit (QIAGEN GmbH, Hilden, Germany). A sequencing library was prepared using a Nextera XT DNA Library Preparation Kit (Illumina, Inc., San Diego, California) and sequenced on a NextSeq 500 (Illumina, Inc., San Diego, California). Pair-end 2 × 150 bp reads were processed by the Tormes pipeline^31^. This whole genome shotgun sequencing project data has been deposited at DDBJ/ENA/GenBank under the accessions listed in Table 5. To reveal phylogenetic relationship between mastitis isolates and *Gordonia* reference genomes^32^, a neighbor-joining tree (Fig. 3) was constructed on the base of presence—absence of genes. Such analysis was done by Roary^33^. Tree was drawn by Mega^34^. For detailed comparison of gene content, the mastitis isolates were compared with the environmental isolates *G. paraffinivorans* NBRC 108238 type strain (genome assembly number GCA_000344155.1, BioSample number SAMD00041809)^9^ and MTZ 041 strain isolated from compost (genome assembly number GCA_003121315.1, BioSample number SAMN03785434)^10^. These genomes were annotated using prokka^35^, and annotation was compared by GenAPI^36^. Virulence factors were identified within the Tormes pipeline by screening the virulence factor database (VFDB)^37^.

**Table 5:** The GenBank assembly accessions number of selected isolates of *Gordonia paraffinivorans.*

Accession number of isolate	Designation of isolate	Source
GCA_012498535.1	G5-ML2190	Milk
GCA_012494195.1	G4-ML1815	Milk
GCA_012494185.1	G3-ML2619/12	Milk
GCA_012498505.1	G2-ML2619/11	Milk
GCA_012498545.1	G1-ML158/2016	Milk
GCA_012498525.1	F12-ML1295/5	Milk
GCA_012498585.1	F11-ML1822/201	Milk
GCA_012498595.1	F10-ML521/4	Milk
GCA_012498665.1	F9-ML765/3	Milk
GCA_000344155.1	NBRC 108238	Environmental
GCA_003121315.1	MTZ 041	Compost

### Antimicrobial susceptibility testing

In all, 21 isolates of *Gordonia* sp. were tested for susceptibility to 7 antimicrobial agents. No more than one isolate collected from the same farm per six-month period was included in antimicrobial susceptibility testing. Disc-based antimicrobial susceptibility testing was performed using Mueller–Hinton agar with sheep blood (TRIOS, spol. s r.o., Prague, CZ) and discs (Oxoid Ltd., Basingstoke, UK) containing penicillin G (10 µg), cefovecin (75 µg), streptomycin (10 µg), neomycin (30 µg), clindamycin (2 µg), tetracycline (30 µg) or cotrimoxazole (25 µg, i.e., 1.25 µg of trimethoprim/23.75 µg of sulfamethoxazole). Interpretation of the results was performed according to clinical breakpoints published in the European Committee on Antimicrobial Susceptibility Testing (EUCAST)—Breakpoint tables for interpretation of MICs and zone diameters^38^ and Comité de l’Antibiogramme de la Société Française de Microbiologie (CASFM)—Recommandations vétérinaires 2018^39^. As interpretation criteria for *Gordonia* sp. do not exist, criteria for *Staphylococcus* spp. or *Corynebacterium* spp. were used (Table 6). To verify the quality of the media and discs, reference strains of *Staphylococcus aureus* (ATCC 25923) and *Escherichia coli* (ATCC 25922) were used. The plates were incubated aerobically at 37 ± 1 °C for 18–24 h.

**Table 6:** Interpretation breakpoint table of zone diameters^a^ used for susceptibility evaluation of *Gordonia* species isolates.

Antimicrobials	Disc content (µg)	Reference species	Susceptibility (Zone in mm)	Resistance (Zone in mm)	Source
Penicillin G	10	*Staphylococcus* spp.	≥ 29	< 29	CASFM, Vet 2018
Cefovecin	30	*Staphylococcus* spp.	≥ 24	< 24	CASFM, Vet 2018
Streptomycin	10	*Staphylococcus* spp.	≥ 15	< 13	CASFM, Vet 2018
Neomycin	30	*Staphylococcus* spp.	≥ 17	< 15	CASFM, Vet 2018
Clindamycin	2	*Corynebacterium* spp.	≥ 20	< 20	EUCAST, 2020
Tetracycline	30	*Corynebacterium* spp.	≥ 24	< 24	EUCAST, 2020
Cotrimoxazole	25	*Staphylococcus* spp.	≥ 16	< 10	CASFM, Vet 2018

^a^Disc diffusion method.

### Ethical approval

Ethical statement is not applicable to this study as the data were gathered through routinely laboratory analysis of samples non-invasively collected from animals on the farms without any animal experimentation. The owners of these animals gave their written permission to use the samples in ongoing studies after the original veterinary use.

## Supplementary Information

Supplementary Legends.

Supplementary Information 1.

Supplementary Information 2.

Supplementary Information 3.

Supplementary Information 4.

## Data Availability

Most data generated and analysed during this study are included in this published article and its supplementary information files (Supplementary files). The sequences of *Gordonia* isolates are freely accessible as GenBank assemblies: https://www.ncbi.nlm.nih.gov/assembly/. Some animal clinical data that support the findings of this study (e.g., names of the farms) are available from State Veterinary Institute Olomouc, Czech Republic, but restrictions apply to the availability of these data, which were used under licence for the current study and so are not publicly available. Data are, however, available from the authors upon reasonable request and with permission of State Veterinary Institute Olomouc, Czech Republic. Short basic description of Czech dairy operations is available in our recent study ^40^.
