# Supplementary material for: Gordonia species as a rare pathogen isolated from milk of dairy cows with mastitis
Source: Sci Rep. 2022 Apr 11;12:6028. doi: 10.1038/s41598-022-09340-4 (PMC9001696; doi:10.1038/s41598-022-09340-4)
Supplement: Supplementary file 1 — Supplementary Legends. [file 41598_2022_9340_MOESM1_ESM.docx]

***Gordonia* species as a rare pathogen isolated from milk of dairy cows with mastitis**

**Jaroslav Bzdil^1^, Sona Slosarkova^2*^, Petr Fleischer^2^, and Jan Matiasovic^2^**

^1^Department of Special Microbiology, State Veterinary Institute Olomouc, Olomouc, 779 00, Czech Republic

^2^Department of Infectious Diseases and Preventive Medicine, Veterinary Research Institute, Brno, 621 00, Czech Republic

*****[slosarkova@vri.cz](mailto:slosarkova@vri.cz)

**Supplementary File Legends**

Supplementary_file_1

Table of MALDI-TOF MS identification scores of *Gordonia* sp. isolates.

Supplementary_file_2

Genome fragments common to all nine selected mastitis isolates but not present in environmental strains.

Supplementary_file_3

Table of genes present in both NBRC 108238 and MTZ 041 genomes but not in mastitis isolates.

Supplementary_file_4

Table of genes present in all mastitis isolates but not in NBRC 108238 and MTZ 041.
