# Supplementary material for: Gordonia species as a rare pathogen isolated from milk of dairy cows with mastitis
Source: Sci Rep. 2022 Apr 11;12:6028. doi: 10.1038/s41598-022-09340-4 (PMC9001696; doi:10.1038/s41598-022-09340-4)
Supplement: Supplementary file 2 — Supplementary Information 1. [file 41598_2022_9340_MOESM2_ESM.docx]

**Supplementary table 1.**

MALDI-TOF MS^1^ identification scores of *Gordonia* sp. isolates

| **Isolate** | **MALDI-TOF MS**  **identification score** | **Genotyping code** |
| --- | --- | --- |
| 1 | 1.868 | F10 |
| 2 | 2.122 |  |
| 3 | 2.135 | F9 |
| 4 | 2.077 | G2 |
| 5 | 2.148 |  |
| 6 | 2.162 | G3 |
| 7 | 1.866 | F12 |
| 8 | 1.833 | G4 |
| 9 | 1.990 |  |
| 10 | 1.879 | G5 |
| 11 | 1.784 | G1 |
| 12 | 1.781 | F11 |
| 13 | 1.772 |  |
| 14 | 1.723 |  |
| 15 | 1.849 |  |
| 16 | 1.806 |  |
| 17 | 1.931 |  |
| 18 | 2.021 |  |
| 19 | 2.319 |  |
| 20 | 1.819 |  |
| 21 | 1.730 |  |
| 22 | 2.000 |  |
| 23 | 1.961 |  |
| 24 | 2.158 |  |
| 25 | 1.761 |  |
| 26 | 1.728 |  |
| 27 | 1.792 |  |

^1^MALDI-TOF MS = Matrix-Assisted Laser Desorption/Ionization coupled to Time-Of-Flight Mass Spectrometry
